# Chirality invertible superstructure mediated active planar optics

**DOI:** 10.1038/s41467-019-10538-w

**Published:** 2019-06-07

**Authors:** Peng Chen, Ling-Ling Ma, Wei Hu, Zhi-Xiong Shen, Hari Krishna Bisoyi, Sai-Bo Wu, Shi-Jun Ge, Quan Li, Yan-Qing Lu

**Affiliations:** 1 National Laboratory of Solid State Microstructures, Key Laboratory of Intelligent Optical Sensing and Manipulation, College of Engineering and Applied Sciences, and Collaborative Innovation Center of Advanced Microstructures, Nanjing University, Nanjing, 210093 China; 2 Institute for Smart Liquid Crystals, JITRI, Changshu, 215500 China; 3 Liquid Crystal Institute and Chemical Physics Interdisciplinary Program, Kent State University, Kent, OH 44242 USA

**Keywords:** Liquid crystals, Applied optics, Optical materials and structures

## Abstract

Active planar optical devices that can dynamically manipulate light are highly sought after in modern optics and nanophotonics. The geometric phase derived from the photonic spin-orbit interaction provides an integrated strategy. Corresponding elements usually suffer from static functions. Here, we introduce an inhomogeneously self-organized anisotropic medium featured by photo-invertible chiral superstructure to realize geometric phase elements with continuously tunable working spectrum and light-flipped phase profile. Via preprograming the alignment of a cholesteric liquid crystal mixed with a photo-responsive chiral dopant, we demonstrate light-activated deflector, lens, Airy beam and optical vortex generators. Their polychromatic working bands are reversibly tuned in an ultra-broadband over 1000 nm covering green to telecomm region. The chirality inversion triggers facile switching of functionalities, such as beam steering, focusing/defocusing and spin-to-orbital angular momentum conversion. This work offers a platform for advanced adaptive and multifunctional flat optics with merits of high compactness, low loss and broad bandwidth.

## Abstract (web summary)

Optically reconfigurable elements are in demand for future applications. The authors report on the use of chirality-invertible cholesteric liquid crystals to actively manipulate geometric phase and create switchable planar optics elements that perform a variety of functions.

## Introduction

Wavefront engineering of light lies in the heart of optics. Along with the miniaturization and integration of modern photonic technology, compact and multifunctional optical devices with active attributes are highly desired. Traditional optical elements rely on the phase accumulation along the propagation, known as the dynamic phase. Spatially curved surfaces or variant refractive indices are inevitable, resulting in a bulky size. Recently developed geometric phase, namely Pancharatnam-Berry phase^1^, derived from the photonic spin-orbit interaction^2^, provides an integrated candidate for functional planar apparatuses^3–5^. It accompanies a space-variant conversion of polarization states and is usually achieved in inhomogeneous anisotropic media^6,7^. Most of such geometric phase elements are static as their functions are fixed once fabricated and hence cannot be varied. Development of actively tunable geometric phase could potentially unlock a variety of advanced adaptive and multifunctional photonic devices, and thus has attracted ever growing attention over the past few years. So far, various strategies have been adopted, such as stretchable substrates^8,9^, controlled chemical reactions^10^, and phase-change materials^11,12^. Nevertheless, it remains a formidable challenge to realize active flat optics with ease of fabrication, convenient operation, high efficiency, on-demand working spectrum and functionalities.

Cholesteric liquid crystal (CLC) is a liquid crystalline phase where the rod-like molecules self-assemble into a helical superstructure with periodic modulation of refractive index, thus forming a one-dimensional soft photonic crystal. Such a superstructure brings in fantastic light manipulation capabilities. The periodically space-variant optical anisotropy of CLC results in a photonic band gap (PBG). Furthermore, owing to its helicity, a spin-selective beam splitting occurs in the band^13^. Circularly polarized light with the same handedness as the chiral structure is Bragg reflected in an equally high efficiency, while the counter one gets totally transmitted. Recently, the spatial modulation of the reflective geometric phase has been reported by controlling the initial alignment of planar CLC^14–16^. Moreover, the intrinsic stimuli-responsive characteristic of this soft matter^17,18^ will endow corresponding elements with the dynamic tunability. The above unique features make such self-organized chiral superstructure a promising choice for active flat optics.

In this work, we introduce a photo-responsive chiral molecular switch and a static dopant with opposite handedness to a liquid crystal host to form a chirality invertible superstructure, thus enabling the active manipulation of geometric phase. Via light stimulation, its helical pitch is continuously tuned and the handedness is reversibly inverted. Accordingly, several active planar optical elements are produced with different photo-patterned CLC superstructures. Ultra-broadband tunable working spectrum and light-triggered function transformation for both Gaussian beams and structured optical fields are verified. This work moves a steady step on the prospective way towards active multifunctional planar optics.

## Results

### PBG tunable and chirality invertible superstructure

**Fig. 1:**
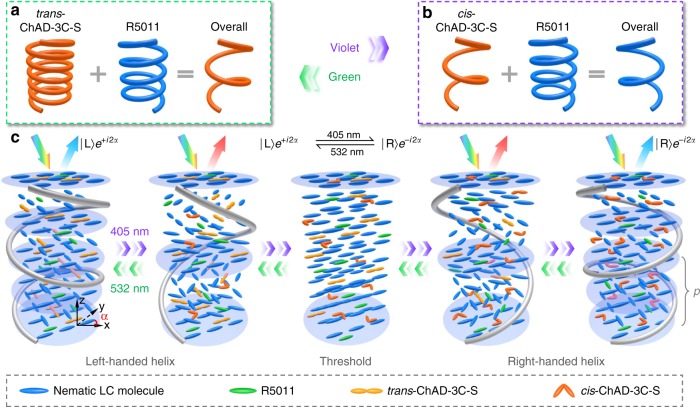
Chirality invertible CLC superstructure directed by light. **a**, **b** Mechanism illustration of the chirality inversion of ChAD-3C-S and R5011 mixture. **c** Schematic illustrations of CLC superstructure evolution driven by the violet (405 nm) and green (532 nm) light. The colorful arrows indicate a white incident light, while blue/red indicates shorter/longer wavelength of Bragg reflected light. *z* depicts the axis of the CLC helix, *α* is the initial orientation angle of local standing helix with respect to *x*-axis, L left circular polarization, R right circular polarization

The light-driven CLC is fabricated by doping a nematic host with a right-handed compound R5011 and a left-handed azobenzene chiral molecular switch ChAD-3C-S, which exhibits excellent chemical stability and good solubility. Upon violet light illumination, the molecules of ChAD-3C-S sequentially isomerize from the rod-like *trans*-form to the bent shaped *cis*-form structures^19^ (see variation of optical absorption spectrum in Supplementary Fig. 1). During this photo-isomerization, its helical twisting power (HTP) drops gradually. In the absence of light stimulus, the opposite *cis*-*trans* isomerization is slow, while it can be drastically accelerated by green light illumination or heating. The effective helical pitch *p* of the prepared CLC can be predicted by

1 $$p = \frac{1}{{c_{\mathrm{S}} \cdot {\mathrm{HTP}}_{\mathrm{S}} + c_{\mathrm{R}} \cdot {\mathrm{HTP}}_{\mathrm{R}}}},$$

where *c*_s_ and *c*_R_ denote the concentrations of ChAD-3C-S and R5011, respectively. The denominator of equation (1) indicates that the overall chirality is a combination of contributions from both chiral dopants. At the initial state, the CLC is left-handed where *trans*-ChAD-3C-S plays a dominant role (Fig. 1a). Under violet light irradiation, HTP_S_ decreases, resulting in a gradual elongation of the helical pitch. When the denominator approaches zero (*p* is infinite), an unwound threshold state is obtained. Further reducing the HTP_S_ leads to a chirality inversion followed by a gradual decrease of *p* (Fig. 1b). Corresponding evolution of CLC superstructure is schematically illustrated in Fig. 1c. Accordingly, a continuously tunable PBG (wavelength range: *n*_o_*p* ~ *n*_e_*p*, *n*_o_/*n*_e_ are the ordinary/extraordinary refractive indices^13^) and optical inversion of chirality could be achieved in a reversible manner.

**Fig. 2:**
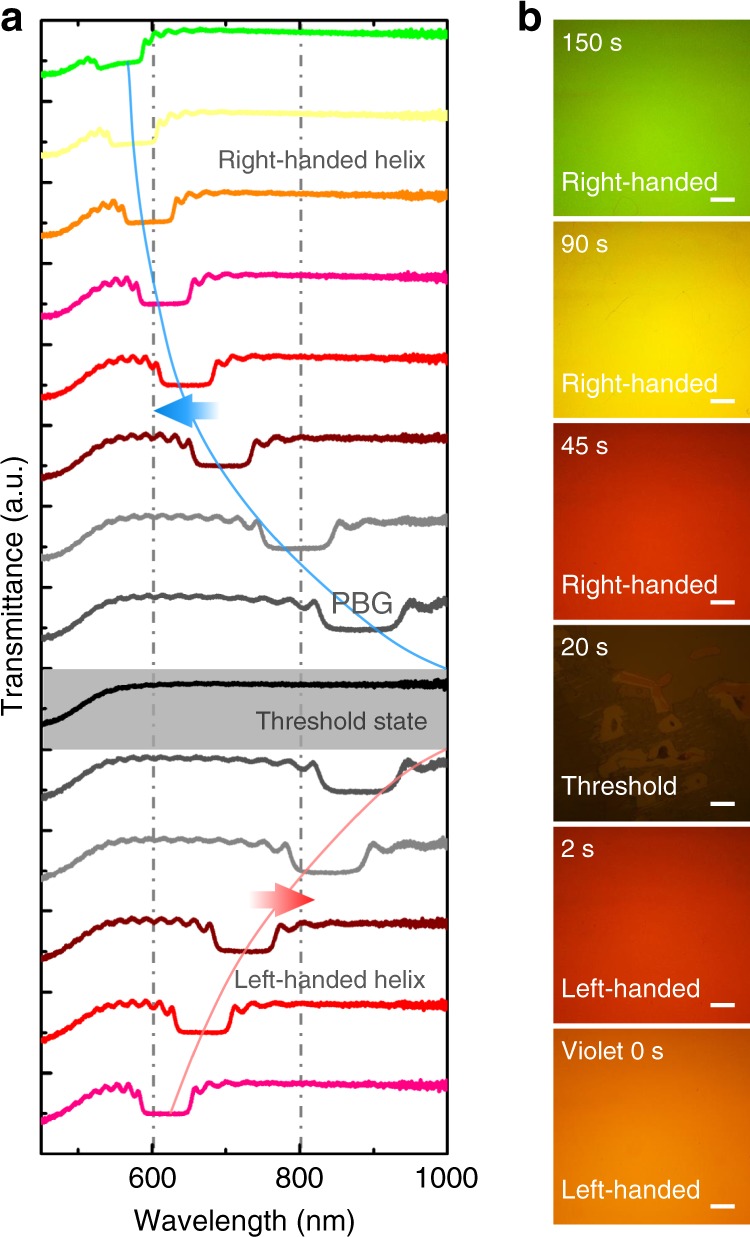
Light-controllable PBG and chirality inversion of CLC superstructure. **a** The transmission spectra of the photo-responsive CLC under violet light irradiation (405 nm, 8.8 mW/cm^2^). **b** Corresponding micrographs observed under the reflective mode of an optical microscope. The light irradiation time and chirality are labelled. All scale bars are 200 μm

The above features are verified in a CLC superstructure as shown in Fig. 2. Stimulated by the violet light, at the beginning, the reflective PBG red-shifts from orange (center wavelength *λ*_c_ = 618 nm) to near-infrared within 20 s. Corresponding CLC forms a typical Grandjean texture exhibiting a large-area uniform and brilliant color consistent with the PBG. At the threshold state (20 s), the PBG disappears owing to the unwinding of CLC. This untwisted phase is featured by the homogeneous state. As it is unstable and transient, some fingerprint textures of a long-pitch CLC are observed as well. Upon further exposure, the CLC transforms to a Grandjean texture again, confirming the chirality inversion of the CLC helical superstructure. PBG re-emerges and blue-shifts to green region (*λ*_c_ = 555 nm). Totally, a PBG variation over 1000 nm is obtained. Besides the vivid visible colors presented in Fig. 2b, the band shift also covers near-infrared range, which is especially interesting for optical telecomm applications. Notably, such chirality inversion and PBG shift are reversible upon alternate irradiation with violet and green light.

### Active planar optics

CLC-based planar optics is realized through spatially modulating the reflective geometric phase. For inhomogeneous CLC, the phase change is twice the initial orientation angle of local standing helix (*α*, Fig. 1c), providing a full control over the reflective phase (0 to 2*π*) by simply rotating *α* from 0° to 180°. Particularly, it exhibits a chirality-dependent sign with + and – indicating left- and right-handedness respectively^20–22^. Here, the chirality inversion induced spin and phase profile conversions can be represented as

2 $$\left| {\mathrm{L}} \right\rangle e^{ + i2\alpha } \mathop{\rightleftharpoons}\limits^{\scriptscriptstyle{405{\mathrm{nm}}}}_{\scriptscriptstyle{532{\mathrm{nm}}}} \left| {\mathrm{R}} \right\rangle e^{ - i2\alpha },$$

where $\left| {\mathrm{L}} \right\rangle$ and $\left| {\mathrm{R}} \right\rangle$ denote left and right circular polarization (LCP/RCP), respectively. For a linearly polarized incident beam, before and after the chirality inversion, equal-energy beams with orthogonal circular polarization would be reflected and endowed with conjugated phase profiles, which is equivalent to the switching of optical functionalities.

**Fig. 3:**
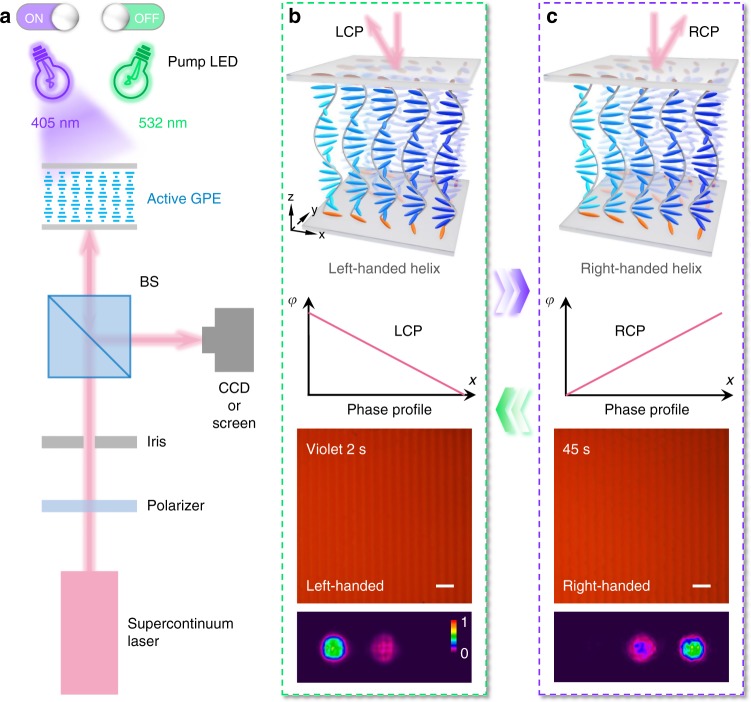
Light-activated beam steering. **a** The optical setup for characterizing active CLC GPEs. CCD, charge-coupled device; BS, non-polarizing beam splitter; GPE, geometric phase element. **b**, **c** Schematic illustrations, theoretical phase profiles, micrographs and reflected diffraction patterns (*λ*_in_ = 650 nm) of the dynamic CLC deflector under (**b**) 2 s and (**c**) 45 s violet light irradiation. The CLC directors on the substrates are highlighted in orange. Note that this system can be reversibly driven by irradiation with green light. Both scale bars are 100 μm. The color bar indicates the relative optical intensity

We employed a photoalignment technique, which is suitable for arbitrary and precise LC patterning^23–25^, to produce inhomogeneous CLC superstructures. A typical photo-switchable beam deflector is demonstrated according to equation (2). Its linearly gradient geometric phase is carried out by a multi-step partly-overlapping photo-exposure process^21,26^ (see details in Supplementary Note 1 and Supplementary Fig. 2). The performance of CLC deflector is characterized with the optical setup illustrated in Fig. 3a. The LCP component of a linearly polarized incident beam within the PBG is endowed with a linearly decreasing phase along *x*, thus being reflected to the left side (Fig. 3b). After chirality inversion, the RCP component experiences a conjugated phase profile and is deflected to the right side (Fig. 3c) while the LCP component gets transmitted. The efficiency of beam steering reaches 76%, which is defined as the intensity ratio of objective order to the total reflection. Such beam deflection is optically reversible thanks to the light-triggered chirality inversion of the CLC superstructure.

**Fig. 4:**
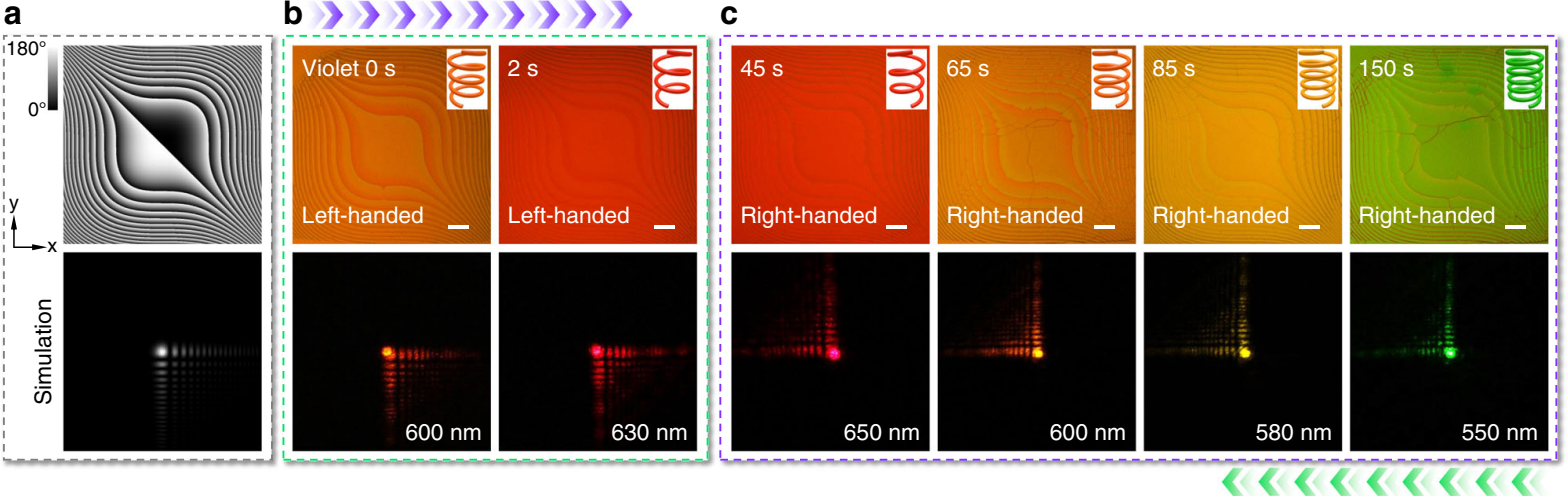
Light-driven spectrum tunable Airy beams. **a** The theoretical *α* distribution of a CLC Airy beam generator and corresponding simulated Airy beam. The color variation from black to white indicates *α* varying from 0° to 180°. **b**, **c** Micrographs and reflected diffraction patterns under 0, 2, 45, 65, 85, and 150 s violet light irradiation, respectively. The chirality of CLC superstructure and wavelength of incident light are labelled. All scale bars are 100 μm

In addition to the dynamic beam deflection and focusing/defocusing (see CLC geometric phase lens in Supplementary Note 2 and Supplementary Fig. 3), more functionalities can be accomplished via rationally programming the chiral superstructures. The past few years have witnessed a sustained interest and impressive progress in structured light research. Airy beam exhibits unique features of non-diffraction, transverse acceleration and self-healing, thus attracting intensive attention^27,28^. It is usually generated through a cubic phase modulation. Accordingly, we introduce a cubically space-variant *α* along both *x*- and *y* axes (Fig. 4a) to the chirality invertible CLC. For such a reflective Airy beam generator, the resultant diffraction patterns are composed of a main lobe and a family of satellite beamlets whose intensity decay exponentially. To explore its photo-tunable polychromatic behavior, we adopted a supercontinuum laser filtered at different monochromatic wavelengths. Along with the light irradiation, uniform colored patterns are clearly observed (Fig. 4b, c), which are consistent with respective PBGs and the predesigned CLC orientation. The generated Airy beams match well with the simulation shown in Fig. 4a. After the chirality is inversed, the transverse accelerating of Airy beam is switched to the opposite direction.

### Light-activated spin-to-orbital angular momentum conversion

**Fig. 5:**
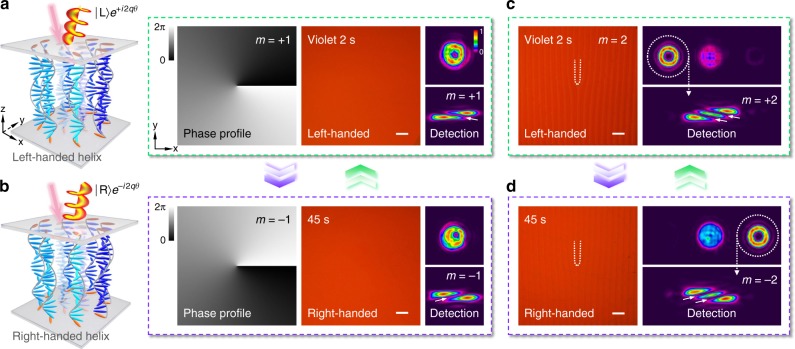
Photo-reversible OAM. **a**, **b** Schematic illustrations, theoretical phase profiles, micrographs, reflected diffraction patterns and corresponding OAM detections of the light-activated CLC *q*-plate with *q* = 1/2 under (**a**) 2 s and (**b**) 45 s violet light irradiation, respectively. The CLC directors on the substrates are highlighted in orange. The color variation from black to white indicates the phase varying from 0 to 2*π*. **c**, **d** Micrographs, reflected diffraction patterns and corresponding OAM detections of the linearly gradient phase integrated CLC *q*-plate (*q* = 1) under (**c**) 2 s and (**d**) 45 s violet light irradiation. All scale bars are 100 μm. The color bar indicates the relative optical intensity

By taking the advantages of this chirality invertible superstructure, unprecedented optical phenomena and properties could be further expected. For instance, the inversion of orbital angular momentum (OAM) is demonstrated in a photo-reversible manner. The helical phase profile of *e*^*imθ*^ induces an OAM of *mћ* per photon^29^, where *m* is the topological charge. Due to the theoretically infinite orthogonal states, OAM beams are a promising candidate for high-capacity optical communications^30^ and high-dimensional quantum informatics^31^. To verify the light-activated OAM inversion, a *q*-plate^32^ is carried out in the chirality invertible CLC with *α* following

3 $$\alpha = q\theta + \alpha _0,$$

where *θ* = arctan(*y*/*x*) is the azimuthal angle, *q* = *m*/2, and *α*_0_ is the initial angle when *θ* = 0 and is usually assumed to be zero. Figure 5a, b schematically illustrate the same CLC *q*-plate (*q* = 1/2) with light-triggered opposite chirality, which induces conjugated phase profiles of *e*^+*iθ*^ and *e*^–*iθ*^. As a result of the central phase singularity, donut-like reflected diffractions are obtained. To detect the topological charges of resultant OAMs, we employed the astigmatic transformation method by inserting a cylindrical lens and capturing the converted pattern at the focal plane^26^. The number of dark stripes and their tilt direction indicate *m* = +1 and *m* = –1.

By further integrating a linearly gradient phase, both functionalities of OAM generation and separation from undesirable components can be achieved simultaneously. An example with *q* = 1 is presented in Fig. 5c, d (see theoretical *α* distribution in Supplementary Fig. 4a). Compared with the CLC deflector (Fig. 3b, c), two forked branches are observed. Due to the chirality inversion of CLC superstructure, OAM beams with opposite topological charge are deflected to symmetric directions. The qualities of generated donut beams are improved significantly, verifying a higher OAM purity. Results with larger *m* are shown in Supplementary Fig. 4b and 4c. Such unprecedented photo-invertible spin-to-OAM conversion enriches the manipulation of light and may inspire versatile applications including reversible optical rotators and steerable qubit in quantum optics.

## Discussion

The performances of the proposed planar optical elements rely simply on the spatial variation of CLC superstructures, and dispense with extra functional materials. Compared with other stimuli (thermal, electric or magnetic field), the light control exhibits superiorities of easy operation, non-invasiveness, and remote spatiotemporal resolution^33–37^. The initial left-handed chiral superstructure is stable due to the high stability of *trans*-ChAD-3C-S in the absence of violet light irradiation. The thermal relaxation of *cis*-state is unavoidable. Fortunately, the helical superstructure could still be maintained for tens of minutes at room temperature under our experimental conditions, after which the PBG (i.e., the working spectrum) would relax back very slowly. This satisfies the requirements of proof-of-principle concept demonstration for potential device applications. By introducing chiral dopants with either low rate of thermal relaxation or absence of thermal relaxation^36–38^, the thermal stability of the devices can be significantly improved. The devices presented here are able to maintain their functions for several weeks. Via further optimizing the package and materials, the durability of these devices could also be extended. The response of these devices reaches ten-second scale under intense-light exposure. By employing more sensitive materials, the response of the devices could be further accelerated.

For visualized illustration, results in visible regime are presented. It is worth noting that the same devices are available in near-infrared region as well. To further expand the PBG, gradient-pitch CLC materials can be employed^39^. Very recently, this strategy has been introduced into the fabrication of ultra-broadband optical elements^40,41^. Besides, light-driven handedness inversion of CLCs has been utilized to achieve rotatable diffraction gratings^35–37^. Despite this progress, the invertible chirality induced functionality transformations and tunable working spectra for structured beams have not been predicted and demonstrated yet. Here, the multi-functionality and reversible performance are achieved in a single device by contactless control using light stimulus. It supplies a universal and practical strategy, simultaneously satisfying two key requirements (switchable functionality and continuously tunable working spectrum) of active planar optics. These cannot be realized with traditional artificial materials. The intrinsic rapid self-organization and high reflection of CLC superstructures make such elements easy-fabrication, cost/energy efficient, and bulk production available. Although the proposed devices cannot process both spin-states at once, a satisfactory solution^22^ can be further introduced to upgrade their functions.

In conclusion, on the basis of a chirality invertible self-organized CLC superstructure, we developed a strategy for active planar optics with unique features of continuously tunable working spectra and light-flipped functionalities. The flexible preprogramming of the chiral superstructures through molecular surface pattern engineering enables on-demand beam tailoring. Various light-driven geometric phase elements such as deflector, lens, Airy beam and OAM generators are presented here. A chirality invertible self-organized chiral system has been exploited in the demonstration of multifunctional planar optical apparatuses with active functionality transformations and tunable working spectra. This work advances the fundamental understanding of soft hierarchical superstructures and lightens holography, optical communications, quantum optics and beyond.

## Methods

### Materials

The photo-responsive CLC was prepared by mixing a commercial nematic LC host HTW114200-050 (HCCH, Δ*n* = 0.222 at 589 nm and 20 °C, clearing point *T*_N-I_ = 107 °C) with 3.1 wt% right-handed chiral dopant R5011 (HCCH, China) and 12 wt% left-handed molecular switch ChAD-3C-S (BEAM, USA). The optical absorption spectra of ChAD-3C-S are presented in Supplementary Fig. 1. R5011 has a relative strong absorption below 350 nm, and its absorption spectrum exhibit no observable change upon violet light irradiation due to the lack of photo-isomerizable groups^42^. The CLC was capillary-filled into a 6 μm-thick sandwich cell with two antiparallel rubbed polyimide layers to explore the light-driven PBGs.

### Sample fabrication

Indium-tin-oxide glass substrates (1.5 × 2 cm^2^) were ultrasonically bathed, UV-Ozone cleaned, and then spin-coated with the polarization-sensitive photoalignment agent SD1 (Dai-Nippon Ink and Chemicals, Japan) dissolved in dimethylformamide (DMF) at a concentration of 0.3 wt%. After curing at 100 °C for 10 min, two pieces of glass substrates were separated by 6 μm spacers and sealed with epoxy glue to form a cell. The empty cell was placed at the image plane of the digital-micro-mirror-device-based microlithography system (Supplementary Fig. 2), and a multi-step partly-overlapping exposure process was performed to carry out the designed *α* distributions accordingly (see Supplementary Note 1 for details). The CLC material was capillary-filled into the photo-patterned cell at 110 °C and slowly cooled to room temperature.

### Characterizations

All experiments were performed at room temperature under ambient environment. The transmission spectra of CLC were measured with a spectrometer (USB4000, Ocean Optics, USA). All micrographs were recorded under the reflective mode of an optical microscope (Nikon 50i, Japan). The supercontinuum fiber laser (SuperK EVO, NKT Photonics, Denmark) was filtered at different monochromatic wavelengths by the multi-channel acousto-optic tunable filter (SuperK SELECT, NKT Photonics). Reflected diffraction patterns from CLC geometric phase elements were captured by a digital camera (EOS M, Canon, Japan) or a CCD camera (BGS-SP620, Ophir-Spiricon, USA).

## Supplementary information

Supplementary Information

## Data Availability

The data that support the findings of this study are available from the corresponding author upon reasonable request.
