# Supplementary material for: Chirality invertible superstructure mediated active planar optics
Source: Nat Commun. 2019 Jun 7;10:2518. doi: 10.1038/s41467-019-10538-w (PMC6555801; doi:10.1038/s41467-019-10538-w)
Supplement: Supplementary file 1 — Supplementary Information [file 41467_2019_10538_MOESM1_ESM.pdf]

Supplementary Information

Chirality invertible superstructure mediated active planar optics

Chen et al.

Supplementary Note 1: Photo-patterning process of CLC chiral superstructures

In this work, a polarization-sensitive sulfonic azo dye SD1 was used as the photoalignment agent, whose molecules tend to reorient their absorption oscillators perpendicular to the polarization direction of the illuminating UV light and further guide LCs[1]. A multi-step partly-overlapping exposure process was performed to carry out the designed α through a digital micro-mirror device (DMD) based dynamic micro-lithography system[2]. The setup is schematically illustrated in Supplementary Fig. 2a. It consists of a light emission component, a dynamic pattern generation component, an image focusing component, and a monitor component. Briefly, a collimated UV light beam is reflected onto the DMD (D4195, Texas Instruments) and carries on a designed pattern. After being focused by a tunable objective, the beam is polarized by a motorized rotating polarizer and then projected onto the empty LC cell. A CCD is utilized to monitor the focusing process. Consequently, arbitrary fine photo-patterned LCs can be obtained.

The α distribution of each CLC element is calculated firstly. Every region with α varying from 0° to 180° is replaced by 36 sub-regions equally. Each sub-region is endowed with a uniform value, from 0° to 175° in the interval of 5°. A sum of five adjacent sub-regions (*i.e.*, the sum-region) is exposed simultaneously with an exposure dose of ~1 J/cm^2, which is not sufficient to induce a stable reorientation of SD1. The subsequent exposure of the sum-region shifts one sub-region with the polarizer rotating 5° synchronously. Finally, each sub-region is exposed five times with a total exposure dose of ~5 J/cm^2, which is sufficient to reorient the SD1 molecules. Three out of all 36 exposure sum-regions of a geometric phase lens and an Airy beam generator, are shown as examples in Supplementary Fig. 2b. Corresponding polarizer angles are listed in Supplementary Fig. 2c. After the 36-step five-time partly overlapping exposure, a quasi-continuous space-variant orientation of SD1 is carried out. After capillary-filled into the photo-patterned cell, the CLC self-assembles into the desired space-variant chiral superstructure, yielding the active geometric phase element.

Supplementary Note 2: Light switchable geometric phase lens

The α distribution of the CLC geometric phase lens follows:

$$\alpha = \frac{\pi}{\lambda}\left(\sqrt{r^2 + f^2} - f\right), \quad (1)$$

where r is the radius, $r^2 = x^2 + y^2$, and f stands for the designed focal length. An example is shown in Supplementary Fig. 3 with f = 12 cm at λ = 650 nm. For left-handed CLC chiral superstructure, the LCP component will be reflected and tailored as a concave parabolic phase profile, leading to a defocused state. While for the violet-light-reversed right-handed CLC lens, the RCP component will be reflected and endowed with a convex parabolic phase, resulting in a focused spot. The direct visualization of these distinct lens functions is shown in Supplementary Fig. 3, which is obtained by observing the reflected patterns on a screen at the distance of 12 cm from the device. Two different irradiation time is chosen to obtain a similar PBG spectrum containing 650 nm. Compared with the reflection from a homogeneously aligned CLC, divergence (a dim background) or convergence (a bright spot) of the reflected light is presented, which are optically switched.

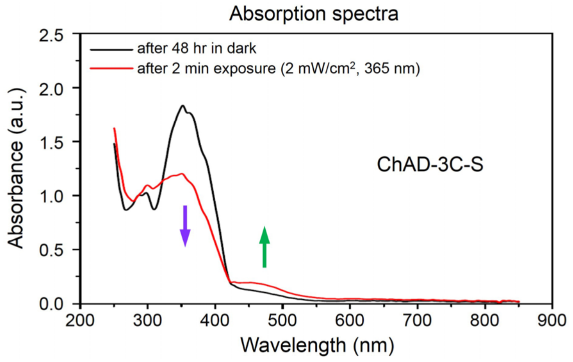

Supplementary Fig. 1 | Variation of optical absorption spectrum of ChAD-3C-S (BEAM, USA) from UV to visible range upon light irradiation.

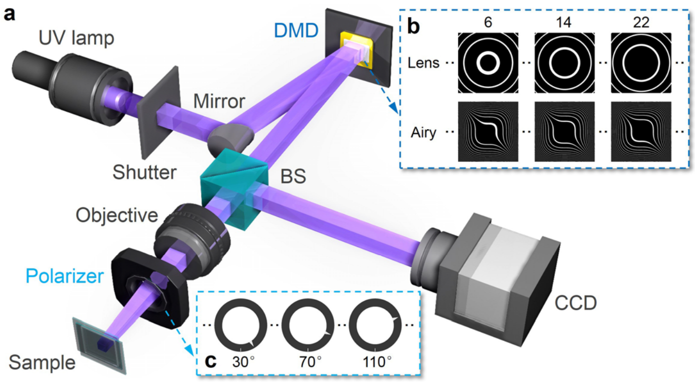

Supplementary Fig. 2 | DMD-based micro-lithography setup. a, The schematic illustration of the setup. **b**, **c**, Three out of all 36 exposure sum-regions from a geometric phase lens and an Airy beam generator with corresponding polarizer angles listed in (**c**).

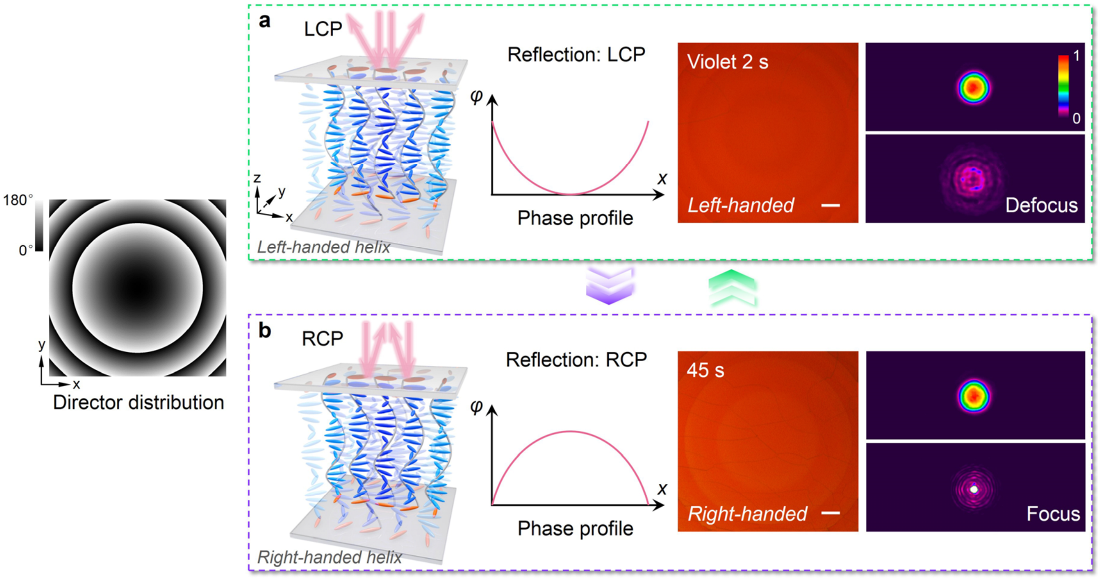

Supplementary Fig. 3 | Light switchable geometric phase lens. The theoretical α distribution is presented on left. The color variation from black to white indicates α varying from 0° to 180°. **a**, **b**, The schematic illustrations, theoretical phase profiles, micrographs and reflected diffraction patterns of CLC lens under (**a**) 2 s and (**b**) 45 s violet light irradiation, respectively. All scale bars are 100 μm. The color bar indicates the relative optical intensity.

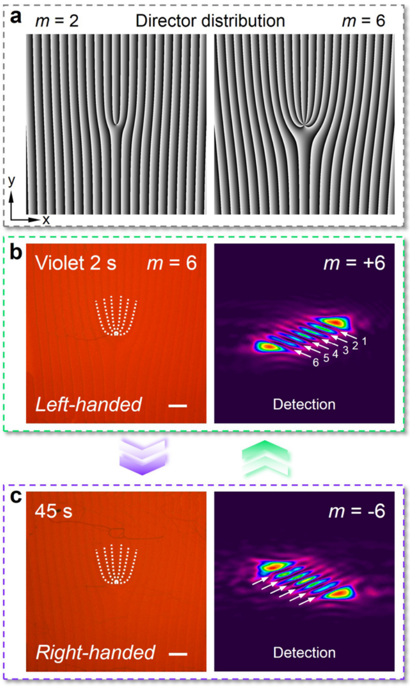

Supplementary Fig. 4 | Light-activated spin-to-orbital angular momentum conversion. **a**, The theoretical α distributions for linearly gradient phase integrated CLC *q*-plates with $m = 2$ and $m = 6$. The color variation from black to white indicates α varying from 0° to 180°. **b**, **c**, Micrographs with $m = 6$ and corresponding OAM detections under (**b**) 2 s and (**c**) 45 s violet light irradiation, respectively. All scale bars are 100 μm.

Supplementary References

1. Chigrinov, V. *et al*. Diffusion model of photoaligning in azo-dye layers. *Phys. Rev. E* **69,** 061713 (2004).
2. Chen, P. *et al*. Arbitrary and reconfigurable optical vortex generation: a high-efficiency technique using director-varying liquid crystal fork gratings. *Photon. Res.* **3,** 133–139 (2015).
